# Síndrome de Brugada: 30 Anos de Aventura Científica

**DOI:** 10.36660/abc.20220289

**Published:** 2023-03-07

**Authors:** Pedro Brugada

**Affiliations:** 1 UZ Brussel Bruxelas Bélgica UZ Brussel , Bruxelas – Bélgica

**Keywords:** Síndrome de Brugada, Morte Súbita, Fibrilação Atrial, Fibrilação Ventricular, Dispositivos Implantáveis

## Abstract

Trinta anos atrás, uma nova síndrome clínico-eletrocardiográfica distinta foi descrita, agora conhecida como síndrome de Brugada (SBr). Típico para essa síndrome é o eletrocardiograma com supradesnivelamento do segmento ST nas derivações precordiais direitas. A apresentação clínica da doença é altamente variável: os pacientes podem permanecer completamente assintomáticos, mas também podem desenvolver episódios de síncope, fibrilação atrial (FA), síndrome do nódulo sinusal (SNS), distúrbios de condução, assistolia e fibrilação ventricular (FV). A doença é causada por mutações nos genes responsáveis pelo potencial de ação das células do coração. O gene mais frequentemente envolvido é o SCN5A, que controla a estrutura e função do canal de sódio cardíaco. A descrição dessa nova síndrome teve implicações muito positivas em todos os campos da medicina.

**Figura Central:**
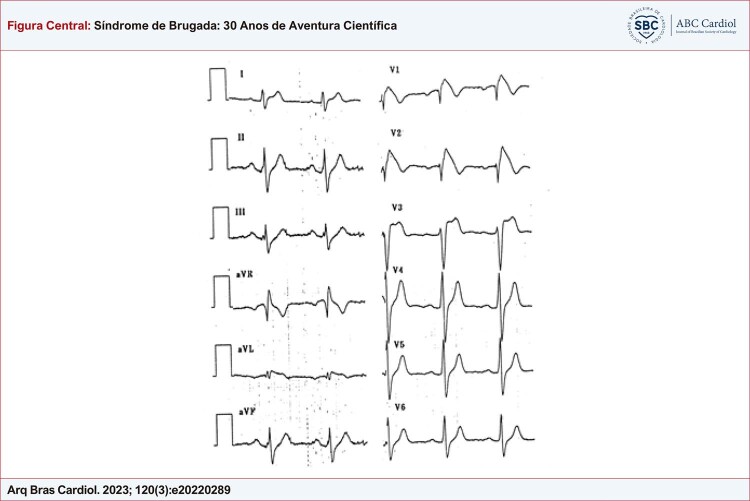
: Síndrome de Brugada: 30 Anos de Aventura Científica

## Introdução

**Figura 1:**
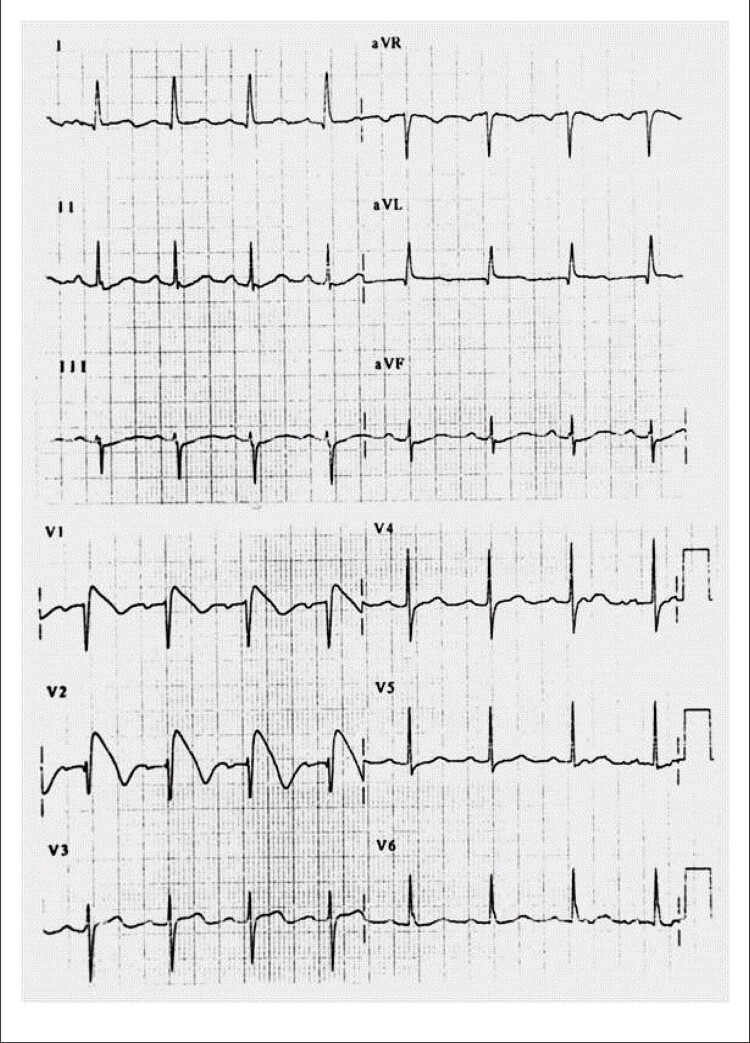
– Eletrocardiograma típico de 12 derivações de SBr. Há supradesnivelamento do ST nas derivações precordiais direitas V1 e V2, com morfologia que lembra a nadadeira de um golfinho.

Em novembro, há 30 anos, o *Journal of the American College of Cardiology* (JACC) publicou um artigo intitulado “Bloqueio do ramo direito, supradesnivelamento persistente do segmento ST e morte súbita: uma síndrome clínica e eletrocardiográfica distinta. Um relatório multicêntrico”. ^1^ Lá, foram descritos 8 pacientes com história de morte súbita ressuscitada causada por FV. Após extensa investigação, nenhuma causa para as arritmias foi encontrada nesses pacientes. Todos os 8 pacientes mostraram um eletrocardiograma muito incomum com supradesnivelamento de ST nas derivações precordiais direitas e o que parecia ser um bloqueio de ramo direito ( Figura 1 ). Três dos pacientes eram crianças. Duas eram meninas. Duas das crianças eram irmão e irmã. Três pacientes também apresentavam características de SSS e três também foram diagnosticados com FA. Quatro pacientes apresentavam distúrbios de condução acentuados e 4 também apresentavam intervalo HV prolongado ou limítrofe, e todos os pacientes apresentavam taquicardia ventricular (TV) polimórfica (sustentada ou não) induzível ao exame eletrofisiológico. As causas da síndrome eram desconhecidas na época, mas ficou imediatamente claro que era um problema hereditário e puramente elétrico do coração - o coração era estruturalmente normal. A FV muito rápida sugeriu um problema de dispersão de períodos refratários curtos ou normais, em contraste com a FV relativamente mais lenta (Torsades de Pointes) da síndrome do QT longo, onde os períodos refratários ventriculares são prolongados devido à repolarização prolongada. A publicação foi o início de uma grande aventura que ainda está em andamento.

Levou 5 anos para coletar os primeiros 4 pacientes. Esses 4 doentes foram apresentados em pôster na reunião da *North American Society of Pacing and Electrophysiology* (Sociedade Norte-americana de Marca-Passo e Eletrofisiologia) - NASPE em 1991. Após a apresentação, e graças à colaboração internacional, foi coletado um grande número de possíveis doentes iguais. Ao final, foram selecionados 4 novos pacientes com características idênticas às dos 4 primeiros. Essa colaboração internacional espontânea (sem financiamento, sem protocolos, sem comitês ou conselhos) resultou em uma das publicações originais mais citadas em cardiologia. O que os autores inicialmente consideraram como uma espécie de curiosidade, mais tarde se tornou uma verdadeira revolução científica. Essa revolução é sentida nas implicações positivas que a descoberta dessa síndrome teve em múltiplos aspectos da medicina.

### Implicações da SBr

**- Para a cardiologia clínica:**

Descrever essa nova síndrome fez uma tremenda justiça ao valor do ECG como um método de diagnóstico simples, barato, mas muito valioso: o diagnóstico da SBr baseia-se no ECG anormal. O eletrocardiograma denominado “tipo 1” ( Figura 1 ) é a única condição para o diagnóstico após a exclusão de outras possíveis causas (fenocópias – Tabela 1 ). A SBr voltou a deixar claro o quanto é perigoso classificar eletrocardiogramas pouco claros como “variantes normais”. O ECG da SBr foi considerado uma variante normal por anos, sem significado diagnóstico ou prognóstico. ^2^ Tivemos que perceber nossa ingenuidade da maneira mais difícil. Tem havido tentativas de estruturar o diagnóstico da SBr por um sistema de pontos. ^3^ Infelizmente, esse escore acaba não tendo valor na prática, pois até 40% dos pacientes com SBr comprovada não teriam critérios suficientes para fazer o diagnóstico por meio desse sistema. ^4^ Pode-se falar de um paciente com ECG de Brugada se não houver outros achados para falar de uma síndrome: como síncope, morte súbita ressuscitada, FA, distúrbios de condução ou mutações patológicas. No momento em que um ou mais desses achados são estabelecidos, pode-se falar de um SBr. A questão é que se pode falar de uma doença de Brugada no momento em que também se encontra uma causa genética da síndrome.

**Tabela 1:** – Fenocópias que podem simular SBr

Drogas antiarrítmicas
Bloqueadores dos canais de sódio classe 1C (por exemplo, flecainida, pilsicainida, propafenona)
Bloqueadores dos canais de sódio de classe 1A (por exemplo, procainamida, disopiramida, cibenzolina)
Verapamil (bloqueador dos canais de cálcio tipo L)
β-Bloqueadores (inibem ICa, L)
**Drogas antianginosas**
Nitratos
Bloqueadores dos canais de cálcio (por exemplo, nifedipina, diltiazem)
**Agentes psicotrópicos**
Antidepressivos tricíclicos (por exemplo, amitriptilina, desipramina, clomipramina, nortriptilina)
Antidepressivos tetracíclicos (por exemplo, maprotilina)
Fenotiazinas (por exemplo, perfenazina, ciamemazina)
Inibidores seletivos da captação de serotonina (por exemplo, fluoxetina)
Intoxicação por cocaína
**Agentes antialérgicos**
Anti-histamínicos histamina H1.
**Isquemia aguda na VSVD**
**Distúrbios eletrolíticos**
Hipercalemia
Hipercalcemia
**Hipertermia e hipotermia**
**Nível elevado de insulina**
**Compressão mecânica de RVOT**

**- Para a fisiologia:**

**Figura 2:**
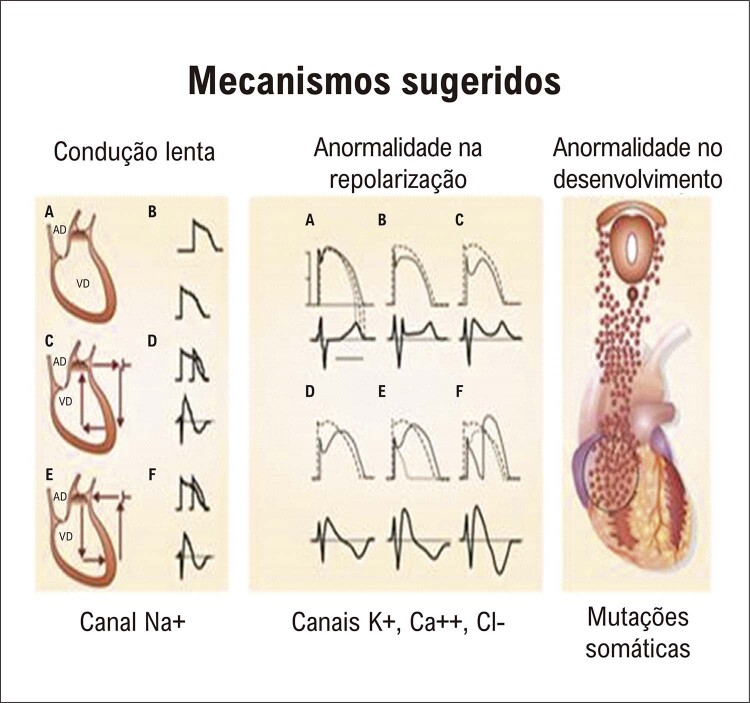
– Ilustração dos três possíveis mecanismos da SBr. O painel A mostra a teoria da despolarização, em que os distúrbios de condução na VSVD deveriam representar o fenômeno mais importante. O painel B mostra o mecanismo de P2R, representando o encurtamento do potencial de ação epicárdico no operatório da VSVD. O painel C mostra a teoria de Elizari, baseada em anormalidades embrionárias na crista neural. O desenvolvimento anormal das células responsáveis pela formação da VSVD com possíveis mutações somáticas seria o problema mais significativo que leva à SBr.

A SBr descobriu novos mecanismos de arritmias, em particular, o fenômeno da “reentrada da fase 2” (RF2) ( Figura 2 ). ^5^ O mecanismo correto da FV na SBr ainda está em debate. Além da reentrada clássica baseada na condução anormal (painel A da Figura 2 ), a RF2 e a teoria da crista neural são duas alternativas para explicar as arritmias. A reentrada clássica na região de saída do ventrículo direito (VSVD) é considerada o mecanismo mais importante para a FV pelo grupo da UMC em Amsterdã. Mas o grupo de Utica se apega à teoria da RF2. Enquanto no primeiro mecanismo os potenciais de ação seriam normais e o gradiente elétrico seria devido à condução lenta com potenciais de ação fora de fase, na RF2 o gradiente elétrico é causado por um encurtamento da duração do potencial de ação no epicárdio da VSVD (painel B). Enquanto no primeiro mecanismo o principal problema residiria em mutações que reduzem o fluxo de sódio na célula cardíaca, a RF2 depende de um fluxo exagerado de potássio (Ito). Curiosamente, mutações na SBr foram encontradas em muitos genes diferentes com uma gama muito extensa de funções. É melhor dizer que a SBr é apenas um fenótipo com possivelmente muitas causas diferentes. É semelhante à síndrome do QT longo (LQT): o ECG mostra um intervalo QT longo, mas as causas podem variar enormemente (íon do canal de sódio no tipo LQT 3, canais de potássio no tipo 1 e 2). O grupo de Elizari, em Buenos Aires, sugere que a base da SBr está em mutações nas células da crista neural que seriam mutações somáticas. Para eles, a SBr é um problema de desenvolvimento do coração na fase embrionária. Interessantemente, mutações nas células germinativas são encontradas em até 40% das famílias com SBr. De fato, é possível que os demais pacientes tenham mutações somáticas que só seriam detectáveis por meio de biópsia da VSVD, e não pelas técnicas usuais, como amostras de sangue. Essa possibilidade foi demonstrada há muito tempo em pacientes com TV idiopática, em que uma biópsia do VD revelou mutações somáticas. ^6^ Essa possibilidade de mutações somáticas na SBr também é corroborada pelo fato de que cerca de metade dos pacientes são casos isolados e não familiares, como se esses pacientes não pudessem transmitir a doença pelas células germinativas.

**- Para a genética:**

A descrição, em 1998, do primeiro gene associado à SBr marcou um verdadeiro ponto histórico nas relações entre genética e cardiologia. Os estudos genéticos em cardiologia eram, portanto, muito limitados e quase exclusivamente focados na busca de mutações em pacientes com LQT ou cardiomiopatia hipertrófica. Os resultados foram vistos mais como uma curiosidade do que uma possível contribuição para entender os mecanismos e, quem sabe, desenvolver um tratamento no futuro. Mas agora podemos começar a entender: o canal de sódio permanece aberto com certas mutações, a repolarização é prolongada e o paciente sofre de LQT (tipo 3). Mas, se a corrente de sódio diminui como resultado de outras mutações no mesmo gene, temos distúrbios de condução e a SBr. De repente, um mundo totalmente novo se abriu. Não é surpresa então que o número de mutações publicadas em todos os distúrbios hereditários cardíacos tenha aumentado muito rapidamente, e não apenas com as doenças antigas e conhecidas. Descobrir o gene responsável pela síndrome do QT curto levou apenas três anos. Com as novas técnicas de pesquisa genética (GWAS), todo o processo de diagnóstico foi acelerado exponencialmente. Infelizmente, todas essas novas informações vêm com um problema de interpretação: todas as mutações e todos os genes realmente importam? Eles são a causa da doença? Qual é a real importância dos polimorfismos? Infelizmente, não temos recursos, tempo e número e variedade suficientes de pacientes para estudar funcionalmente cada mutação, para mostrar que os efeitos esperados de uma mutação específica correspondem ao que esperamos como manifestação da doença. Existem modelos para nos ajudar, mas os resultados dos modelos sempre vêm com um grau de probabilidade e incerteza sobre o valor do resultado. No entanto, esses resultados são importantes para o tratamento de famílias com SBr.

**- Para a fertilidade:**

Com todas as limitações que se possa imaginar, o diagnóstico genético pré-implantar (DGP) tornou-se uma opção óbvia para o tratamento de doenças hereditárias. Os opositores da técnica argumentam que quase nenhuma doença, especialmente a SBr, é monogenética. Segundo eles, além do principal gene considerado responsável pela doença, deve haver uma série de outras mutações e variações, inclusive polimorfismos, que se acumulam até que um certo “escore de risco genético” seja alcançado. Assim, implantar um embrião selecionado com base, por exemplo, na ausência de mutação no canal de sódio, não teria valor na prevenção da SBr. Os defensores do DGP dizem, com os mesmos argumentos, que apenas selecionar um embrião sem a mutação diminui esse escore de risco genético e, assim, podemos neutralizar a manifestação da doença. O DGP é oferecido em nosso hospital há anos para mais de 200 doenças diferentes que são consideradas monogenéticas, incluindo a SBr. Dada a pouca idade das crianças nascidas após o DGP, ainda não podemos tirar conclusões sobre se elas desenvolvem ou não a doença. Essas crianças e jovens são monitorados de perto.

Outro aspecto da fecundidade diz respeito aos abortos espontâneos em famílias com SBr. Embora seja possível que embriões e fetos tenham morrido “in utero” de arritmias, ou mesmo de o coração nunca bater, é impossível tirar conclusões. ^7^ Existem muitos abortos espontâneos que acontecem completamente despercebidos em mulheres férteis para estudar esse fenômeno.

**- Para a ginecologia:**

Dadas as graves consequências da SBr, não é surpresa que as pessoas tenham se perguntado quais poderiam ser as consequências da doença para as mulheres grávidas. As informações coletadas mostram que a gravidez e o parto não apresentam riscos particulares ^7^ em mulheres com SBr.

**- Para a pediatria:**

A SBr é uma causa de morte súbita em crianças, e também uma das várias causas possíveis da síndrome da morte súbita infantil (SMSI). Poucas doenças foram especuladas por tanto tempo e tão esotericamente como no caso da SMSI. Desde a postura do bebê ao dormir, fumar no quarto do bebê, usar ou não usar travesseiros, e mais, as mais diversas explicações não científicas eram constantemente buscadas. Agora sabemos que a gama de causas da SMSI é muito ampla e que, de fato, asfixia infantil, assassinato e acidentes ocultos podem desempenhar um papel na morte. Mas agora também sabemos que a maioria dessas mortes súbitas são devido a arritmias, incluindo a SBr. ^8^

Encontramos um problema semelhante no diagnóstico de epilepsia e síncope de causa desconhecida em crianças. Não só o LQT, mas também a síndrome do QT curto e a SBr devem ser incluídas no diagnóstico diferencial, e ainda mais se a criança tiver síncopes ou epilepsia “difíceis de tratar”. Também não devemos esquecer que os pacientes também podem sofrer de mais de uma doença: epilepsia e SBr juntos ^9^ e também síncope vasovagal e simultaneamente arrítmica.

**- Para a farmacologia:**

Durante anos, muitas pesquisas foram feitas em relação às alterações do intervalo QT no ECG devido a drogas. Um grupo de trabalho internacional está fazendo uma atualização quase semanal de medicamentos que podem prolongar o intervalo QT. ^10^ Um prolongamento do intervalo QT pode resultar no desenvolvimento de “Torsade de Pointes” e morte súbita. Agora sabemos, graças aos efeitos das drogas na SBr, que outras drogas também podem causar morte súbita por seus efeitos no canal de sódio. Uma lista desses medicamentos também é mantida ativa por um consórcio internacional. ^11^

**- Para a medicina esportiva:**

Nada afeta mais nossa imaginação do que a morte súbita de um atleta “perfeitamente saudável”. Consideramos essas pessoas as mais saudáveis da nossa sociedade e é quase impossível compreender que possam morrer inesperadamente. Isso também acontece com atletas profissionais que são monitorados anualmente quanto a doenças cardiovasculares. Nem toda morte súbita em atletas ocorre durante o exercício. Na verdade, o contrário é verdadeiro. ^12^ A maioria morre subitamente após o esforço, imediatamente ou mais tarde, em repouso completo. Sabe-se há muito tempo que a síndrome do LQT e a TV polimórfica dependente de catecolaminas (TVPC) eram uma causa. Mas agora, após exame detalhado dos parentes do falecido, parece que a causa mais frequente é a SBr. ^12^ O fato de a SBr poder apresentar um ECG completamente normal torna extremamente difícil a detecção desses pacientes.

**- Para a medicina legal:**

Não existem boas regras em muitos países em relação à realização de autópsias. No caso da morte súbita de um jovem, esta pesquisa dificilmente é levada em conta. A autópsia só se tornará obrigatória se for uma morte não natural. Os resultados dessas autópsias variam enormemente de estudo para estudo, bem como de acordo com a experiência dos médicos e sua “resistência”. Um médico procura mais e mais profundamente pela causa da morte do que outro. Mas, mesmo após o que é chamado de “autópsia especializada”, um grupo muito grande de pacientes permanece sem diagnóstico. ^12^ Aqui que entram em jogo o estudo dos membros da família e a “autópsia molecular”. O estudo de Papadakis et al. ^12^ mostrou que a causa mais frequente de morte súbita – quando a causa é encontrada – é a SBr. Isso foi demonstrado por eles pelos resultados do teste de ajmalina nos membros da família. Testes genéticos *post-mortem* também podem revelar uma possível mutação causadora em 20 - 40% dos casos. ^13^

**- Para a medicina preventiva:**

A triagem de pessoas que parecem saudáveis é uma das melhores maneiras de descobrir doenças ocultas. Mas é claro que o valor da triagem está muito relacionado ao pesquisador e aos estudos que estão sendo conduzidos. Todos nós aceitamos a triagem de mulheres para câncer de mama ou a detecção de potenciais portadores de tumores de cólon que ainda não se manifestaram. Mas, quando se trata de triagem cardiovascular, as opiniões divergem. Infelizmente, os argumentos a favor e contra a triagem não parecem tão difíceis. Se olharmos para os resultados da prevenção do câncer colorretal na Holanda, parece que 95% dos testes positivos – suspeitos – são falsos positivos. Assim, 95% dos “pacientes” fazem uma colonoscopia por nada. ^14^ Isso também vale para o rastreamento do câncer de mama. Os resultados são tão controversos que as autoridades médicas na Suíça pararam de rastrear o câncer de mama. Quanto ao coração, as conclusões dependem das pesquisas que acreditamos: a favor do rastreamento, como sugerem os estudos italianos ^15^ ou contra o rastreamento, como sugerem os estudos americanos. Há, em todo caso, uma grande diferença nos argumentos a favor ou contra. Enquanto os italianos baseiam seus argumentos na redução da incidência de morte súbita graças à triagem, os argumentos americanos contra a triagem são puramente financeiros. Mas, que preço damos à vida de um jovem?

**- Para medicina do trabalho:**

Talvez a contribuição mais extraordinária para a descrição do SBr seja a compreensão de alguns dos “mistérios” médicos. Tomemos, por exemplo, a alta incidência na década de 1970 de morte súbita na América de trabalhadores do sul da Ásia. Não havia como entender por que esses jovens assintomáticos e de aparência saudável morreram repentinamente. Dada a presença quase endêmica da SBr no sul da Ásia, e após as investigações de Nademannee, ^16^ sabemos agora que a SBr foi a causa da morte. Isso foi comprovado não apenas por estudos clínicos, mas também por estudos genéticos. ^17^

**- Para a anestesia:**

Na lista de medicamentos que podem causar morte súbita em pacientes com SBr, encontramos também os anestésicos. ^10^ Um deles é o propofol, responsável pela chamada síndrome do propofol. ^18^ Essa síndrome também está relacionada à SBr. ^19^ Curiosamente, em um de nossos estudos, não encontramos inconvenientes com o uso de propofol em pacientes com SBr comprovada. ^20^ As manifestações da síndrome de propofol podem depender da dose, da sensibilidade do paciente, mas outras patologias subjacentes certamente também desempenham um papel. A síndrome de propofol manifesta-se com alargamento muito bizarro do complexo QRS no ECG com elevações típicas de ST compatíveis com SBr e com arritmias ventriculares que podem causar morte súbita do paciente.

**- Para a medicina de emergência:**

A SBr é parte obrigatória do diagnóstico diferencial de vários problemas médicos: síncope, trauma e acidentes de trânsito causados por uma possível arritmia ou comprometimento temporário da consciência, epilepsia, todas as formas de parada cardíaca e arritmias ventriculares, distúrbios de condução. Um grupo que deve ser discutido separadamente são os pacientes jovens com FA ou flutter atrial. Antes de injetar drogas intravenosas para parar essas arritmias, deve-se sempre perguntar se isso não poderia ser SBr. A administração de flecainida intravenosa em tal patente pode levar à morte.

**- Para os dispositivos eletrônicos cardíacos e ablação da VSVD:**

**Figura 3:**
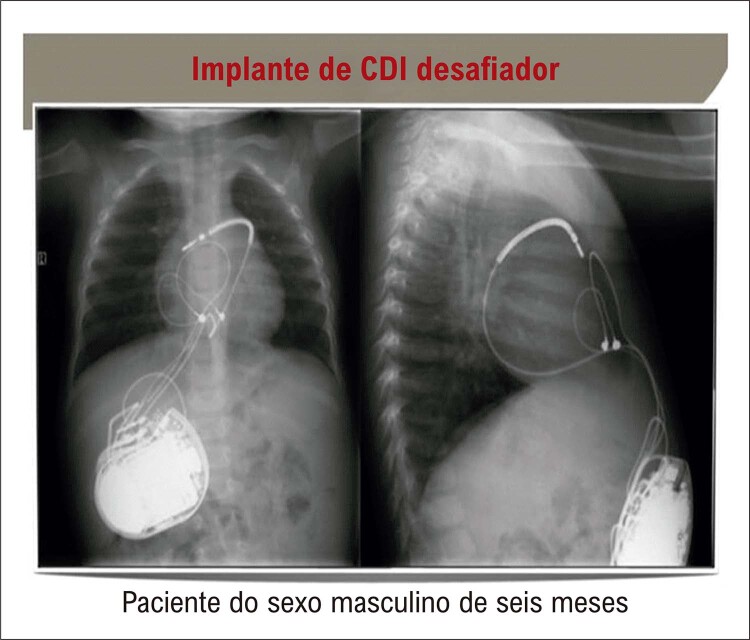
– Radiografia de tórax do implante abdominal de um desfibrilador (CDI) em um bebê de 6 meses. O eletrodo de choque está localizado no seio transverso com o eletrodo distal aparafusado no átrio direito. Dois eletrodos epicárdicos são colocados para detecção e estimulação na parede livre do ventrículo direito. O sistema é completamente extravascular com todos os benefícios de um CDI DDD. Algoritmos de discriminação, detecção e estimulação DDD e estimulação antitaquicardia estão, em contraste com o CDI subcutâneo, totalmente disponíveis.

SBr é uma doença de jovens. Só pode ser controlada (em termos de morte súbita) com a implantação de um desfibrilador interno (CDI). Não é surpresa, então, que as técnicas de implantação tenham sido adaptadas às crianças. Por exemplo, um implante abdominal subcostal é muito mais confortável do que um implante pré-peitoral, principalmente no que diz respeito à prática esportiva. Com o treinamento necessário, o implante pode ser feito com fios epicárdicos, de modo que o sistema venoso do paciente seja totalmente poupado ( Figura 3 ). Em centros experientes, esse implante epicárdico pode ser combinado com uma ablação epicárdica da VSVD, onde está localizado o substrato para a SBr. ^21^ Essa combinação é o nosso protocolo atual para o tratamento de SBr. Deve ficar claro que ainda não temos dados suficientes sobre os efeitos a longo prazo da ablação. Atualmente, a ablação não é uma alternativa ao CDI.

**- O mundo da morte súbita reimaginado:**

Entramos em um novo mundo de causas de morte súbita em pessoas com coração estruturalmente normal: de QT longo após SBr como a principal causa. ^12^

**- Aspectos de gênero:**

Muitas publicações sobre SBr sempre enfatizaram que os homens com essa doença se saem pior que as mulheres. Embora esses pensamentos pareçam verdadeiros em adultos, eles não o são antes da puberdade. Nenhuma diferença foi encontrada nos sintomas e mortalidade entre meninos e meninas pré-púberes. ^22^ É muito claro que a testosterona desempenha um papel na SBr. A própria castração masculina demonstrou melhorar as manifestações da doença. ^23^

**- Para entender certas tradições folclóricas:**

As tradições sem linguagem são agora muito melhor compreendidas depois de descrever o SBr. Na Tailândia, por exemplo, os homens se vestem como mulheres depois de se casarem quando vão para a cama com a noiva. Diz a tradição que uma bruxa fica com ciúmes quando uma jovem se casa e como castigo vem sufocar o noivo à noite. Lai Tai é o nome dado a essa morte súbita na Tailândia, pois a vítima começa a roncar antes da morte. Pokkuri no Japão e Bangungut nas Filipinas referem-se ao mesmo fenômeno.

**- Para a história:**

Existem inúmeros casos de morte súbita na história que, como Lai Tai e Pokkuri, ficaram inexplicáveis. Claro que é impossível ter certeza se a SBr teve algum papel nisso, mas é no mínimo interessante especular sobre isso. Veja o caso de Tommy Morris, indiscutivelmente um dos melhores golfistas de sua época (ele venceu o Open aos 17 anos). Tommy foi encontrado morto em sua cama pela manhã aos 24 anos. Ele não tinha queixas antes da morte. Pelo contrário, ele tinha acabado de jogar e ganhou um jogo de golfe de 200 buracos (!) na neve. Duas possíveis causas para a morte foram sugeridas: uma hemorragia interna e um “coração partido”, pois sua esposa havia morrido durante o parto com a criança alguns meses antes. Uma morte súbita à noite aos 24 anos certamente nos sugere uma possível SBr.

Um segundo caso interessante é o conhecido cantor Michael Jackson. Ele faleceu subitamente aos 50 anos após uma injeção de propofol. A SBr no diagnóstico diferencial é mais do que apropriada.

**- Para a medicina animal:**

A morte súbita não é estranha ao mundo animal. ^24^ Uma busca sistemática das possíveis causas nunca foi feita. Só recentemente, após intensa análise desse fenômeno, a SBr foi incluída na lista de possíveis causas.

**- Para a filosofia: Paradoxos e a SBr:**

**Figura 4:**
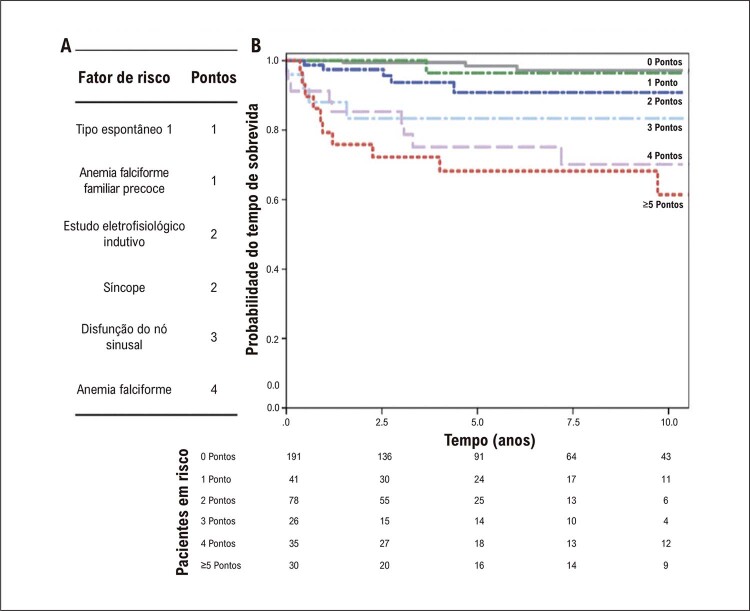
– Sistema de pontuação de Sieira et al. À esquerda os fatores de risco são mostrados com os pontos de valor para cada parâmetro. O gráfico mostra o risco de morte súbita (ressuscitada) durante um período de acompanhamento de 10 anos, dependendo dos pontos.

**Figura 5:**
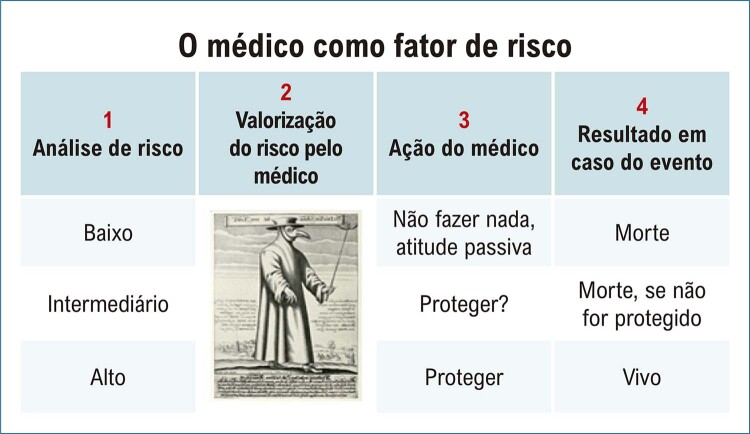
– O paradoxo da estratificação de risco.

Um aspecto muito interessante da SBr diz respeito à avaliação do risco de morte súbita. Conforme relatado na introdução, a SBr tem uma gama muito ampla de apresentações clínicas. O diagnóstico pode ser feito após um episódio de morte súbita ressuscitada, mas há cada vez mais pacientes diagnosticados e completamente assintomáticos. Síncope, FA, SNS, distúrbios de condução são sintomas e achados com impacto no prognóstico. Mas a pergunta é: quem deve fazer o CDI preventivamente? Metade dos pacientes que sofreram morte súbita não apresentavam sintomas antes da morte súbita. A outra metade teve uma síncope ou pré-síncope em algum momento. Somente após extensa pesquisa o Prof. Sieira chegou a uma “pontuação” para estimar o risco de morte súbita ^25^ ( Figura 4 ). Quanto mais pontos o paciente obtiver, maior será o risco de parada cardíaca no futuro. Esse sistema de estratificação de risco é muito valioso, mas carrega um enorme paradoxo: ao classificar os pacientes em grupos de baixo, médio e alto risco, podemos cometer o erro de considerar que o baixo risco significa ausência de risco. Pacientes com escore baixo, portanto, não são considerados candidatos à proteção com CDI. Por outro lado, pacientes com pontuação alta são sistematicamente protegidos. O resultado, paradoxalmente, é que os pacientes de alto risco e proteção pelo CDI sobrevivem às crises de arritmias graças à proteção do CDI, enquanto no grupo de baixo risco, se ocorrer uma arritmia, resultará em morte por falta de proteção com um CDI. Assim, embora a incidência de arritmias seja muito menor na categoria de baixo risco, a mortalidade real é maior devido à falta de proteção. Esse paradoxo é ilustrado na Figura 5 .

## Conclusão

Em 30 anos aprendemos muito sobre a SBr, mas também sobre outras doenças relacionadas. É claro que, com a descrição da SBr, todo o mundo da Ritmologia entrou em uma nova dimensão.
