# Supplementary material for: Síndrome de Brugada: 30 Anos de Aventura Científica
Source: Arq Bras Cardiol. 2023 Mar 7;120(3):e20220289. [Article in Portuguese] doi: 10.36660/abc.20220289 (PMC12080723; doi:10.36660/abc.20220289)
Supplement: Translation

# Translation: Brugada Syndrome: 30 Years of Scientific Ventures

**Authors:** Pedro Brugada

**Affiliations:** 1 Bélgica UZ Brussel, Bruxelas – Bélgica

**Keywords:** Brugada Syndrome, Sudden Death, Atrial Fibrillation, Ventricular Fibrillation, Implantable Devices

## Abstract

Thirty years ago, a distinctly new clinical-electrocardiographic syndrome was described, today known as Brugada Syndrome (BrS). Typical treatment for this type of syndrome is electrocardiography with ST-segment elevation in the direct precordial derivations. The clinical presentation of the disease is highly variable: the patients can remain completely asymptomatic, but they can also develop episodes of syncope, atrial fibrillation (AF), sinus node dysfunction (SNF), conduction disorders, asystole, and ventricular fibrillation (VF). This disease is caused by mutations in the genes responsible for the potential action of cardiac cells. The most commonly involved gene is SCN5A, which controls the structure and function of the heart’s sodium channel. The description of this new syndrome has shown highly positive implications in all fields of medicine.

**Central Illustration:** : Brugada Syndrome: 30 Years of Scientific Ventures

## Introduction

In November, 30 years ago, the Journal of the American College of Cardiology (JACC), published an article entitled: “Right bundle branch block, persistent ST-segment elevation, and sudden death: a distinct clinical and electrocardiographic syndrome. A multicenter report”,^1^which described eight patients with a medical history of resuscitated sudden death caused by ventricular fibrillation (VF). After extensive investigation, no cause for the arrythmias was found in these patients. All of the eight patients showed a highly uncommon electrocardiography with ST-segment elevation in the direct precordial derivations and what appeared to be a right bundle branch block ( Figure 1 ). Three of the patients were children; two were girls; and two of the children were brother and sister. Three patients also presented characteristics of SSS and three were diagnosed with AF. Four patients present accentuated conduction disorders and four presented a prolonged HV intervals or limits, and all of the patients presented polymorphic (sustained or not) ventricular tachycardia (VT), susceptible to the electrophysiological exam. The causes of the syndrome were unknown at the time, but it became evidently clear that there was a hereditary and purely electric problem of the heart – the heart was structurally normal. The extremely quick VF suggested a problem of the dispersion of short or normal refractory periods, as compared to the relatively slow VF (Torsades de Pointes) of the Long QT Syndrome, where the refractory ventricular periods are extended due to the prolonged repolarization. The publication was the beginning of a major scientific venture that is still ongoing.

**Figure 1:** – Typical electrocardiogram of 12 derivations of BrS. There is an ST-segment elevation in the direct precordial derivations, V1 and V2, with a morphology that looks like the fin of a dolphin.

It took five years to collect data from the first four patients. These four patients were shown in a poster presentation at the North American Society of Pacing and Electrophysiology Meeting (NASPE) in 1991. After the presentation, and thanks to international collaboration, data from four new patients with characteristics identical to the first four were collected. This spontaneous international collaboration (without financing, without protocols, without committees or councils) resulted in one of the most cited original publications in cardiology. What the authors initially considered as a type of curiosity, later transformed into a scientific revolution. This revolution can still be seen in the positive impacts that the discovery of this syndrome has had on multiple aspects of medicine.

### BrS impacts

**- For clinical cardiology:**

The description of this new syndrome was of great value for ECG as a simple, inexpensive, yet valuable diagnostic method: The BrS diagnosis is based on the abnormal ECG. The “type 1” electrocardiogram ( Figure 1 ) is the only condition for the diagnosis after the exclusion of other possible causes (phenocopies – Table 1 ). The BrS once again made it clear just how dangerous it can be to classify fairly unclear electrocardiograms as “normal variants”. The BrS ECG was considered to be a normal variant for years, with no significant diagnosis or prognosis.^2^We had to realize our own naivety the hard way. There have been attempts to structure the diagnosis of BrS using a point system.^3^Unfortunately, this score actually has no value in practice, since up to 40% of the patients with diagnosed BrS would not have sufficient criteria to reach this diagnosis using this system.^4^A patient can be diagnosed with BrS ECG if there are no other findings to refer to a syndrome: such as syncope, resuscitated sudden death, conduction disorders, or pathological mutations. At the moment in which one or more of these findings are established, one can provide a diagnosis of BrS. The question at hand is if one can still claim a diagnosis of Brugada disease at the time in which the genetic cause of the syndrome is identified.

**Table 1:** – Phenocopies that can simulate BrS

Antiarrythmic drugs
Sodium channel blockers, class 1C (for example, flecainide, pilsicainide, propafenone)
Sodium channel blockers, class 1A (for example, procainamide, disopyramide, cibenzoline)
Verapamil (Calcium channel blockers, type L)
β-Blockers (inhibit ICa, L)
**Antiaginal drugs**
Nitrates
Calcium channel blockers (for example, nifedipine, diltiazem)
**Psychotropic agents**
Tricyclic antidepressants (for example, amitriptyline, desipramine, clomipramine, nortriptyline)
Tetracyclic antidepressants (for example, maprotiline)
Phenothiazine (for example, perphenazine, cyamemazine)
Selective inhibitors of the uptake of serotonin (for example, fluoxetine)
Cocaine intoxication
**Antiallergy agents**
Antihistamine H1
**Acute RVOT ischemia**
**Electrolytic disorders**
Hypercalemia
Hypercalcemia
**Hyperthermia and hypothermia**
**High insulin level**
**Mechanical compression of RVOT**

*RVOT: right ventricular outflow tract.*

**- For Physiology:**

The BrS discovered new mechanisms of arrythmias, in particular, the “2^nd^stage re-entry” phenomenon (RF2) ( Figure 2 ).^5^The correct mechanism of VF in BrS is still under debate. In addition to the classic re-entry, based on an abnormal conduction (Panel A of Figure 2 ), the RF2 and the neural crest theory are two alternatives to explain arrythmias. The classic re-entry in the right ventricular outflow tract (RVOT) is considered to be the most important mechanism for VF, according to the UMC group in Amsterdam. However, the Utica group clings more to the RF2 theory. While in the first mechanism, the action potential would be normal and the electric gradient would be due to the slow conduction with out-of-phase action potentials, in RF2, the electric gradient is caused by a shortening of the duration of the action potential in the epicardium of the RVOT (panel B).

**Figure 2:** – Illustration of the three possible mechanisms of BrS. Panel A shows the depolarization theory, in which the conduction disorders in the RVOT should represent the most important phenomenon. Panel B shows the P2R mechanism, representing the reduction of the action potential of the epicardium in the RVOT operation. Panel C shows the Elizari theory, based on embryonic abnormalities in the neural crest. The abnormal development of the cells responsible for the formation of the RVOT with possible somatic mutations would be the most significant problem that could lead to BrS.

While in the first mechanism the main problem resides in the mutations that reduce the flow of sodium in the heart cells, the RF2 depends on an exaggerated flow of potassium (Ito). Curiously, the mutations in the BrS were found in many different genes, with a highly extensive array of functions. It is better to say that the BrS is only a phenotype, possibly with many different causes. It is similar to the Long QT (LQT) syndrome in that the ECG shows an LQT interval, but the causes can vary greatly (ion of the sodium channel in LQT type 3 and sodium channels in types 1 and 2). The Elizari group, in Buenos Aires, suggests that the basis of BrS is in the mutation in the neural crest cells, which would be somatic mutations. For these, the BrS is a development problem of the heart in the embryonic stage. Interestingly enough, mutations in the germinative cells are found in up to 40% of the families with BrS. In fact, it is possible that the other patients have somatic mutations that would only be detectable through a RVOT biopsy and not through the usual techniques, such as blood samples. This possibility has been demonstrated for some time in patients with idiopathic VT, in which a biopsy of the RV revealed somatic mutations.^6^This possibility of somatic mutations in the BrS is also corroborated by the fact that nearly half of the patients are isolated cases and not family oriented, as if these patients were unable to transmit this disease through germinative cells.

**- For genetics:**

The description, in 1998, of the first gene associated with BrS was a true benchmark in the history of relations between genetics and cardiology. The study of genetics in cardiology was, therefore, highly limited and almost exclusively focused on the search for mutations in patients with LQT or hypertrophic cardiomyopathy. The results were viewed more as a curiosity than a possible contribution to the understanding of the mechanisms, which could quite possibly develop into a future treatment. But today we can begin to understand the following: the sodium channel remains open, with certain mutations; the repolarization is prolonged; and the patient suffers from LQT (type 3). However, if the sodium channel decreases as a result of other mutations in the same gene, we then have conduction disorders and BrS. Suddenly, a whole new world has been opened. It is thus no surprise that the number of publications on mutations in all of the hereditary heart disorders have increased quickly, and not only with the age-old and well-known diseases. Discovering the gene responsible for the short QT syndrome took only three years. With the new techniques of genetic research (GWAS), the entire diagnostic process was sped up exponentially. Unfortunately, all of this new information comes with one problem of interpretation: Do all of the mutations and all of the genes really matter? Are they the cause of disease? What is the true importance of polymorphisms? Unfortunately, we do not have the resources, time, nor number and variety enough of patients to functionally study each mutation so as to show that the likely effects of a specific mutation correspond to what we expect to be the manifestation of the disease. There are models that can help us, but the results of the models always come with a degree of probability and uncertainty concerning the value of the result. Nevertheless, these results are important for the treatment of families with BrS.

**- For fertility:**

With all of the imaginable limitations, the preimplantation genetic diagnosis (PGD) has become an obvious option for the treatment of hereditary diseases. Opposers to the technique argue that almost no disease, especially BrS, is monogenetic. According to them, in addition to the main gene considered to be responsible for the disease, there is also likely to be a series of other mutations and variation, including polymorphisms, which accumulate until a certain “genetic risk score” is achieved. Therefore, implanting an embryo selected based on, for example, the absence of mutation in the sodium channel would have no value in the prevention of BrS. Using the same arguments, advocates of PGD claim that merely selecting an embryo without the mutation would diminish this genetic risk score, thereby neutralizing the manifestation of the disease. The PGD has been provided in our hospital for years to combat more than 200 different monogenetic diseases, including BrS. Given the young age of the children born after PGD, it is still impossible to draw conclusions about whether they developed the disease or not. These children and youth are being monitored closely.

Another aspect of fertility refers to miscarriages in families with BrS. Although it is possible that embryos and fetuses have died “in utero” from arrythmias, or even due to a heart that never beat, it is still impossible to draw conclusions.^7^Many miscarriages happen in fertile women and go unnoticed, which needs to be studied in greater detail.

**- For gynecology:**

Of the severe consequences of BrS, it is no surprise that individuals have asked themselves what might be the possible consequences of the disease for pregnant women. The gathered information shows that the pregnancy and the birth present no particular risks^7^in women with BrS.

**- For pediatrics:**

The BrS is one sudden cause of death among children, as well as one of the many possible causes of Sudden Infant Death Syndrome (SIDS). Few diseases have been speculated for so long and so esoterically as in the case of SIDS. From the posture of the baby when sleeping, to smoking in the baby’s bedroom, to using pillows or not, among others, the broadest variety of non-scientific explanations has constantly been searched for. We now know that the array of causes of SIDS is quite broad and that, in fact, child asphyxia, murder, and hidden accidents can play a role in the child’s death. But we also know now that most of these sudden deaths are due to arrythmias, including BrS.^8^

We have found a similar problem in the diagnosis of epilepsy and syncope of unknown cause in children. Not only the LQT, but also the short QT syndrome and the BrS, should be included in the differential diagnosis, and even more if the child has types of syncope or epilepsy that are “difficult to treat”.

We should also remember that the patients can suffer from more than one disease: epilepsy and BrS together,^9^as well as vasovagal syncope and simultaneous arrythmia.

**- For pharmacology:**

For years, many studies have been conducted on the alterations in the QT interval in the ECG due to drugs. One international working group is currently conducting an almost weekly updating of the medications that can prolong the QT interval.^10^One extension of the QT interval can result in the development of “Torsade de Pointes” and sudden death. We now know that, thanks to the effects of drugs on BrS, other drugs can also cause sudden death due to their effects on the sodium channel. One list of these medicines has also been kept active by an international consortium.^11^

**- For sports medicine:**

Nothing affects our imagination more than the sudden death of a “perfectly healthy” athlete. We consider these people to be the healthiest individuals in society, and it is almost impossible to understand that they can die unexpectedly. This also occurs with professional athletes who are monitored yearly regarding cardiovascular diseases. Not all sudden deaths among athletes happens during exercise. In fact, the contrary is actually true.^12^Most die suddenly after stress, immediately or after a complete resting period. It has been known for some time that the LQT syndrome and the catecholaminergic polymorphic VT (CPVT) was a cause. But now, after a detailed examination of the parents of the deceased, it seems that the most common cause is BrS.^12^The fact that BrS can present a completely normal ECG makes it extremely difficult to detect in these patients.

**- For forensic medicine:**

Proper rules to conduct autopsies are lacking in many countries. In the case of the sudden death of a young individual, this type of study is rarely taken into account. The autopsy will only be mandatory if it is an unnatural death. The results of these autopsies vary greatly from study to study, as well as according to the experience of the doctors and their “resistance”. One doctor will search more and more in-depth about the cause of death than will another. However, even after what is called a “specialized autopsy”, a large group of patients still remains undiagnosed.^12^What is important here is the study of the family members and the “molecular autopsy”. Findings from Papadakis et al.^12^showed that the more frequent cause of sudden death – when the cause is found – is BrS. This was demonstrated by the results of the Ajmaline test conducted with family members. Post-mortem genetic tests can reveal a possible causal mutation in 20-40% of the cases.^13^

**- For preventive medicine:**

The screening of individuals who appear to be healthy is one of the newest ways to discover hidden diseases. However, it is clear that the value of the screening is heavily related to the researcher and to the studies being conducted. We all accept the screening of women for breast cancer or the detection of individuals prone to contract colon tumors but who have not yet manifested any symptoms. However, with cardiovascular screening, the opinions diverge. Unfortunately, the arguments for and against screening do not seem to be so difficult. If we look at the results from colorectal cancer prevention in the Netherlands, it appears that 95% of the suspected positive tests turn out to be false positives. Hence, 95% of the “patients” do a colonoscopy for no reason.^14^This is also valid for the screening of breast cancer. The results are so controversial that the medical authorities in Switzerland have stopped screening for breast cancer. Concerning the heart, the conclusion depends on studies that we believe to be in favor of screening, as suggested by studies conducted in Italy.^15^or against screening, as suggested by studies conducted in America. There is, in any case, a major difference in the arguments in favor and against. While the Italians base their arguments on the decrease in the incidence of sudden death due to screening, the American arguments against the screening are purely financial. But what price should we put on the life of a young individual?

**- For occupational health:**

The most extraordinary contribution to the description of BrS may well be the understanding of some of the medical “mysteries”. We take, for example, the high incidence in the 1970s of sudden death in America of Southern Asian workers. There was not way to know why these asymptomatic young individuals, with an apparently healthy appearance, died so suddenly. Given the almost endemic presence of the BrS in Southern Asia, and after the Nademannee investigations,^16^we now know that BrS was the cause of death. This was proven by both clinical trials and genetic studies.^17^

**- For anesthesia:**

In the list of medications that can cause sudden death in patients with BrS, we also came across anesthetics.^10^One of these is propofol, responsible for the so-called propofol syndrome.^18^This syndrome is also related to BrS.^19^Curiously, in one of our studies, no inconveniences were found with the use of propofol in patients with proven BrS.^20^The manifestations of the propofol syndrome can depend on the dose or the sensitivity of the patient, but other subjacent pathologies can also play a role. The propofol syndrome appears with a bizarre expansion of the QRS complex in the ECG with typical ST-segment elevations that are compatible with BrS and with ventricular arrythmias that can cause the patient’s sudden death.

**- For emergency medicine:**

BrS is an integral part of the differential diagnosis of many medical problems: syncope, trauma, and traffic accidents caused by a possible arrythmia or temporary loss of consciousness, epilepsy, all forms of heart attacks and ventricular arrythmias, and conduction disorders. One of the groups that should be discussed separately is that of young AF or atrial flutter patients. Before injecting intravenous drugs to stop arrythmias, one should always ask if this might be BrS. The administration of intravenous flecainide in this scenario could lead to death.

**- For the pacemakers and RVOT ablation**

BrS is a disease of young individuals. It can only be controlled (in terms of sudden death) with an implantable cardioverter defibrillator (ICD). Thus, it comes as no surprise that the implantation techniques have been adapted for children. For example, a subcostal abdominal implant is much more comfortable than a pectoral implant, mainly as regards sports practices. With the necessary training, the implant can be made with epicardial strands in such a way that the venous system of the patients is fully spared ( Figure 3 ). In experienced centers, this epicardial implant can be combined with an RVOT epicardial ablation, where the BrS substrate is located.^21^This combination is our current protocol for the treatment of BrS. It is important to note that we do not yet have sufficient data on the long-term effects of the ablation. Currently, the ablation is not an alternative to the ICD.

**Figure 3:** – Chest X-ray of the abdominal implant of an ICD in a 6-month old baby. The chock electrode is located in the transverse sinus with the distal electrode screwed into the right atrium. Two epicardial electrodes are placed to detect and stimulate the free wall of the right ventricle. The system is completely extravascular with all of the benefits of an ICD DDD. Algorithms of DDD discrimination, detection, and stimulation, as well as anti-tachycardia stimulation, in contrast to the subcutaneous ICD, are fully available.

**- The world of sudden death re-imagined:**

We are entering a new world of causes of sudden death in individuals with structurally normal hearts, with Long QT after BrS as the main cause.^12^

**- Gender aspects:**

Many publications on BrS have always emphasized that men with this disease are more heavily affected than are women. Although these findings seem to be true in adults, they are not true before puberty. No difference was found in the symptoms between prepubertal boys and girls.^22^It is quite clear that testosterone plays a role in BrS. Male castration itself showed improvements in the manifestations of the disease.^23^

**- To understand certain folklore traditions:**

The traditions without language are now much more well-understood after having given a proper description of BrS. In Thailand, for example, men dress up as women after getting married when they go to bed with their bride. According to the tradition, a witch becomes jealous when a young woman marries and, as punishment, she comes to suffocate the groom at night. Lai Tai is the name of this sudden death in Thailand, as the victim begins to snore before his death. Pokkuri in Japan and Bangungut in the Philippines refer to the same phenomenon.

**- For history:**

There are innumerous cases of sudden death in history that, as happens in Lai Tai and Pokkuri, are unexplainable. Of course, it is impossible to be sure if BrS played a key role in this, but it is at least interesting to speculate about it. Take the case of Tommy Morris, unquestionably one of the best golfers of his day (he won the Open at 17). Tommy was found dead in his bed in the morning at 24. He had no complaints before his death. To the contrary, he had just finished playing and won a 200-hole golf game (!) in the snow. Two possible causes for this death were suggested: internal hemorrhaging and a “broken heart”, as his wife had died when giving birth to their child only a few months earlier. A sudden death at night at 24 years of age certainly suggests a possible BrS.

A second interesting case is that of the well-known singer, Michael Jackson, who died suddenly at 50 years of age after an injection of propofil. BrS in the differential diagnosis would be more than appropriate in this case.

**- For animal medicine:**

Sudden death is not strange in the animal world.^24^A systematic search for possible causes was never conducted. Only recently, after an intense analysis of this phenomenon, was the BrS included in the list of possible causes.

**- For philosophy: Paradoxes and the BrS:**

One very interesting aspect of the BrS refers to the evaluation of the risk of sudden death. According to that reported in the introduction, BrS has a broad array of clinical presentations. The diagnosis can be performed after an episode of resuscitated sudden death, but there have been more and more diagnosed and asymptomatic patients. Syncope, AF, SNF, and conduction disorders are symptoms and findings with an impact on the prognosis. But the question is: Who should do an ICD preventively? Half of the patients who suffer from sudden death presented no symptoms before sudden death. The other half had a syncope or pre-syncope at some point. Only after an extensive study did Professor Sieira reach a “score” to estimate the risk of sudden death^25^( Figure 4 ). The more points the patient had, the greater the risk of heart failure in the future. This risk stratification system is quite valuable, but it also carries an enormous paradox: upon classifying patients in groups of low, medium, and high risk, we can commit the error of considering that the low risk means the absence of risk. Patients with a low score, therefore, are not considered candidates for protection with ICD. By contrast, patients with a high score are systematically protected. The result, paradoxically, is that the high-risk patients protected by ICD tend to survive crises of arrythmias thanks to the ICD protection, whereas in the low-risk group, if there is an arrythmia, it will likely result in death due to a lack of ICD protection. Therefore, although the incidence of arrythmias is much lower in the low-risk category, the true mortality is higher due to the lack of protection. This paradox is illustrated in Figure 5 .

**Figure 4:** – Sieira et al. scoring system. To the left, the risk factors are shown with the points of value for each parameter. The graph shows the risk of sudden death (resuscitated) during a 10-year follow-up period, depending on the score.

**Figure 5:** – The paradox of risk stratification.

## Conclusion

In 30 years, much has been learned about BrS, as well as about other related diseases. It is clear that, with the BrS description, all professionals from the field of Clinical Arrythmology have now entered a new dimension in the field.
